# Noncontact and instant detection of phosphor temperature in phosphor-converted white LEDs

**DOI:** 10.1038/s41598-017-18686-z

**Published:** 2018-01-10

**Authors:** Tsung-Hsun Yang, Hsu-Yi Huang, Ching-Cherng Sun, Benoît Glorieux, Xuan-Hao Lee, Yeh-Wei Yu, Te-Yuan Chung

**Affiliations:** 1 Department of Optics and Photonics, National Central University, Chung-Li, Taoyuan City, 32001 Taiwan; 2 Optical Sciences Center, National Central University, Chung-Li, Taoyuan City, 32001 Taiwan; 3 ICMCB-CNRS, 87, Av. Du Dr Albert Schweitzer, 33608 PESSAC Cedex, France

## Abstract

Phosphor-converted white light-emitting diodes (pc-WLEDs) have become a major light source in general lighting. To stabilize the photometric characteristics of pc-WLEDs, much effort has been made to manage the heat dissipation of the LED dies. The thermal problems of the phosphor parts, a critical reliability concern for pc-WLEDs, have recently attracted academic interest. This study proposed a practical approach for measuring phosphor temperature in an operating pc-WLED using a noncontact, instant detection method to remotely monitor the emission spectrum. Conventionally, an infrared camera or thermocouples have been used to measure temperature. An IR camera requires good calibration on the emissivity and is usually blocked by the lens or other components covered on the phosphors. Moreover, a thermocouple requires time to reach the thermal equivalence between the detector and the sample under testing, and this approach is destructive when used for inner detection. Our approach has advantages over the conventional methods because it is noninvasive, noncontact, and instant, and inner detection. The approach is also independent of the peak wavelength of pumping lights, the concentration and thickness of phosphor, and correlated color temperatures.

## Introduction

**Figure 1:**
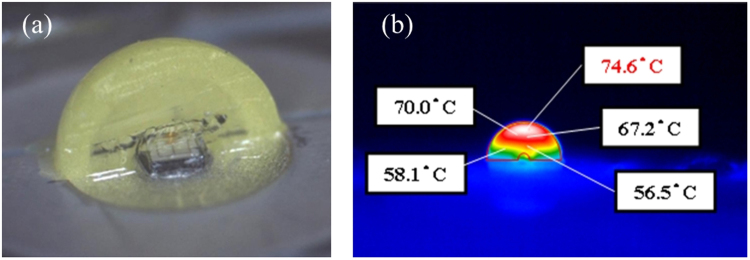
(**a**) Vertical cross-section of an LED packaged in a blue LED die covered with yellow phosphor dome. One-half of the phosphor dome was removed for the temperature distribution detection using IR camera (**b**) Detected temperature distribution.

**Figure 2:**
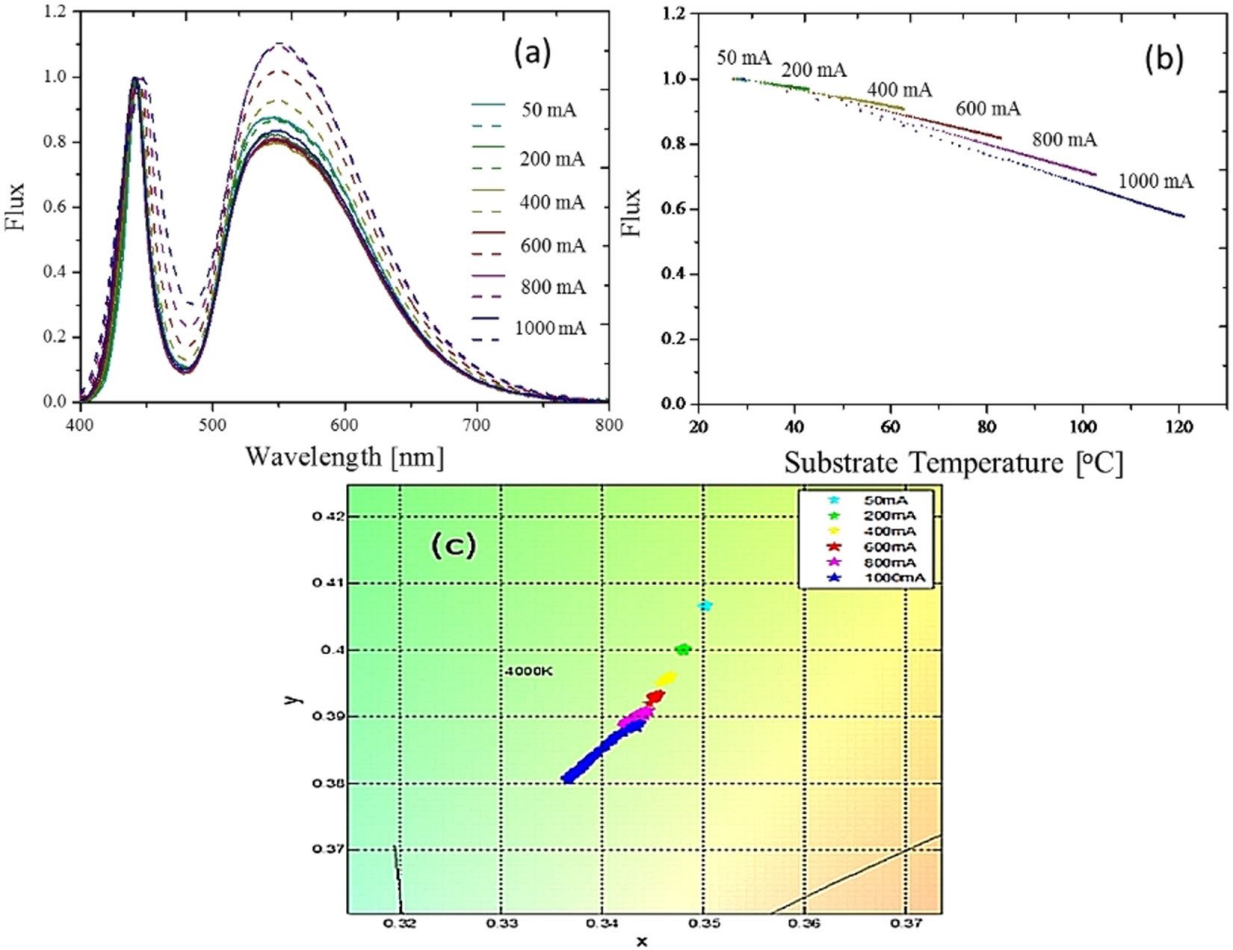
(**a**) Temperature-dependent normalized spectra with different injection currents. Solid line: initial stage, dash line: steady state. (**b**) Variation of the normalized power corresponding to different injection currents. (**c**) Corresponding drift in color coordinates.

Solid-state light sources have played a major role in modern lighting in both outdoor and indoor applications. Among the lighting applications, phosphor-converted white light-emitting diodes (pc-WLEDs) are a key light source because they are inexpensive, fast, and efficient; have strong color performance; and have acceptable reliability. Generally, a pc-WLED contains a yellow (or green with red) phosphor layer that covers a blue die to emit white light in a wide correlated color temperature (CCT) range with an acceptable color rendering index^1–5^. The performance of a pc-WLED can be judged by the efficiency, color accuracy, and reliability^6^. The reliability is related to heat flow and temperature distribution in the package volume^7–10^. In our previous study, we found that the hottest spot in the package volume was located at the top of the blue chip or in the phosphor layer^11^ (a package with remote phosphor) (Fig. 1). High temperature in the package volume of a pc-WLED can cause thermal quenching of phosphor, resulting in color drift, which induces blue-light leakage, efficiency droop, and lifetime shortening^12–14^ (Fig. 2). Thus, managing the thermal effect is critical in extending the lifetime of a pc-WLED. Generally, the junction temperature of the LED die is considered an indicator of the thermal condition in both academia and industry^15–21^. However, the junction temperature of the LED die is not strongly correlated with the temperature in the phosphor layer. Thus, more research is needed to understand temperature in the phosphor layer and package volume^22^. Conventionally, three categories–invasive, semi-invasive, and noninvasive–have been used to describe the different techniques for measuring the material temperature^23,24^. Invasive measurements are impractical for pc-WLEDs because of the difficulties in destroying the completeness of the packaging and those associated with the long time required for thermal equivalence at the thermal contacts. In semi-invasive measurements, temperature-sensitive materials are introduced, which modify the component of interest and therefore disturb the temperature field. Finally, noninvasive measurements are a type of indirect spectroscopic approach^25–28^. This approach monitors either the intensity shifts or wavelength shifts of the fluorescence lines, which are highly sensitively to phosphor temperature. Measuring the wavelength shifts is usually more accurate than measuring the intensity shifts; however, both of these noninvasive approaches require specially designed phosphors for the temperature sensors. These types of temperature-sensitive phosphors degrade the stability of pc-WLEDs in most applications. Even when applying an infrared (IR) camera using infrared thermography, the temperature on the surface of samples is primarily measured, rather than the temperature inside the samples. No effective method has yet been developed for measuring the phosphor temperature in pc-WLEDs. So far, the thermal dynamics of the phosphor layer/volume in pc-WLEDs still lacks useful support.

This paper proposes a novel noninvasive method for building a database for a specific phosphor with a specific LED bonding that has been proven to detect phosphor temperature precisely for different pumping blue lights and different phosphor concentrations or CCTs.

## Results

Because of space constraints, inserting a thermal sensor into the phosphor layer was difficult. In addition, the sensor absorbed the blue or yellow light and generated heat itself. A passive method to measure the temperature of a pc-WLED is needed.

### Analysis of Thermal-dependent Spectra

**Figure 3:**
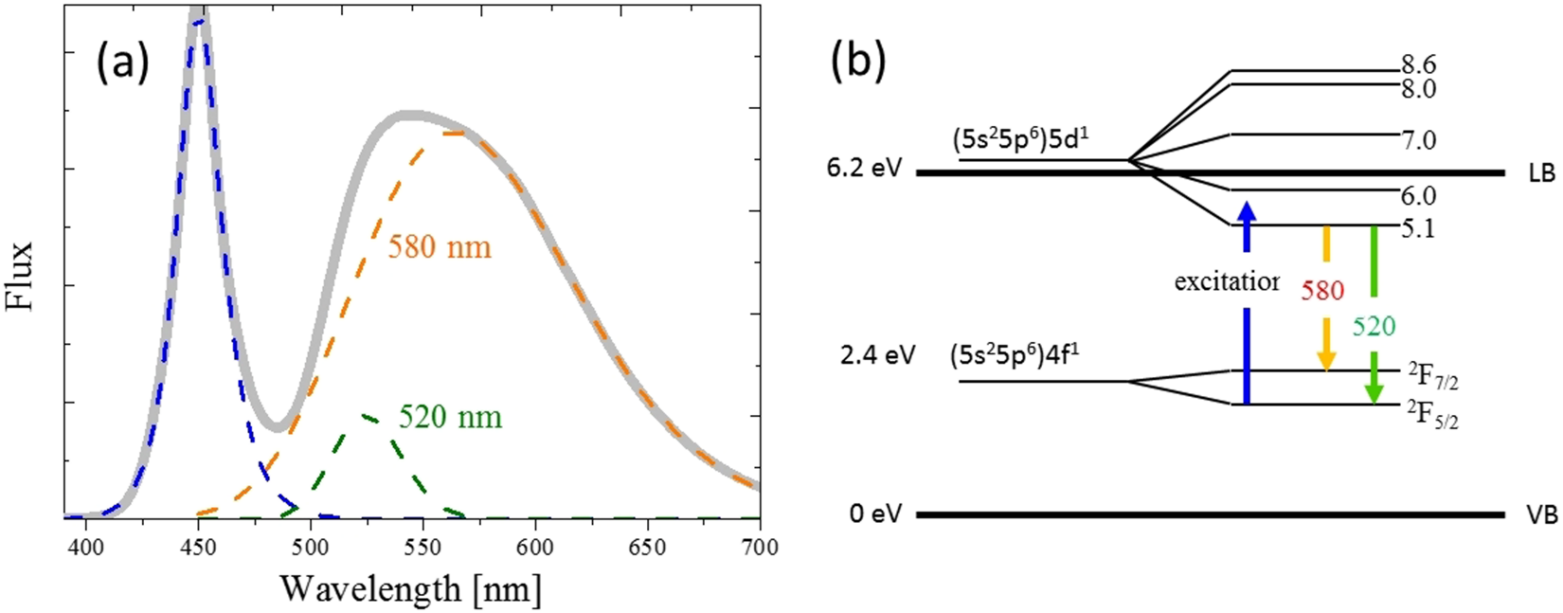
(**a**) Normalized emission spectrum of phosphor consisting of the transition between two subbands. (**b**) Two subbands corresponding to two specific down conversions.

Variations in the emission spectra of the phosphors indicated the phosphor temperature, but a useful signal must be extracted from the noises and overlapping phenomena. Figure 3 shows the normalized emission spectrum of YAG: Ce^3+^, which was a yellow phosphor with the maximum peak at approximately 540 nm. From the band structure, the down conversion comprised two separate Gaussian contributions in the energy range due to the spin orbit coupling effect between the (5*s* ^2^5*p* ^6^) 5*d* ^1^ excited state and (5*s* ^2^5*p* ^6^) 4*f* ^1^ ground state. The two subbands corresponded to the ${}^{2}T{}_{2g}-{}^{2}F_{\mathrm{7/2}}$ and ${}^{2}T{}_{2g}-{}^{2}F_{\mathrm{5/2}}$ transitions^29^, with a respective maximum at 580 nm (called long band) and 520 nm (called short wavelength band)^12,30^.

This means that the entire down conversion spectrum shown in Fig. 3 was composed of two subspectra. As reported in an earlier study^31^, the blue spectrum can be fitted using Eq. (1) quite well:

1 $${F}_{b}(\lambda )=\frac{2{A}_{b}}{{e}^{-\frac{(\lambda -{\lambda }_{b})}{{\sigma }_{l}}}+{e}^{\frac{(\lambda -{\lambda }_{b})}{{\sigma }_{r}}}},$$

where *A*_*b*_, *λ*_*b*_, *σ*_*l*_, and *σ*_*r*_ are the strength, peak wavelength, short bandwidth, and long bandwidth of the blue light, respectively. The yellow spectrum emitted by the phosphor is well fitted by the sum of two asymmetrical spectral functions as each shown in Eq. (2):

2 $${F}_{p}(\lambda )={A}_{p}\frac{{\lambda }_{p}^{2}}{{\lambda }^{2}}{e}^{-\frac{{(\lambda -{\lambda }_{p})}^{2}}{{(\lambda \cdot {\sigma }_{p})}^{2}}},$$

where *A*_*p*_, *λ*_*p*_, and *σ*_*p*_ represent the strength, peak wavelength, and corresponding bandwidth of the asymmetrical spectral function, respectively. The asymmetrical spectral function form, *F*_*p*_(*λ*), was converted from a Gaussian form as a function of *v*(≡C/*λ*)^32^ based on the condition of the equal energy between the intervals in terms of *dv* and of *dλ*. Therefore, the total spectrum of the pc-WLED, *F*_*T*_(*λ*), can be expressed as the sum of a blue LED spectrum (*F*_*b*_(*λ*)) and two phosphor spectra of a short wavelength band (*F*_*pS*_(*λ*)) and a long wavelength band (*F*_*pL*_(*λ*)), as shown in Eq. (3).

3 $${F}_{T}(\lambda )={F}_{b}(\lambda )+{F}_{pS}(\lambda )+{F}_{pL}(\lambda )=\frac{2{A}_{b}}{{e}^{-\frac{(\lambda -{\lambda }_{b})}{{\sigma }_{l}}}+{e}^{\frac{(\lambda -{\lambda }_{b})}{{\sigma }_{r}}}}+{A}_{pS}\frac{{\lambda }_{pS}^{2}}{{\lambda }^{2}}{e}^{-\frac{{(\lambda -{\lambda }_{pS})}^{2}}{{(\lambda \cdot {\sigma }_{pS})}^{2}}}+{A}_{pL}\frac{{\lambda }_{pL}^{2}}{{\lambda }^{2}}{e}^{-\frac{{(\lambda -{\lambda }_{pL})}^{2}}{{(\lambda \cdot {\sigma }_{pL})}^{2}}}\mathrm{.}$$

In the experiment, we attached a blue die to a metal core printed circuit board (MCPCB) and covered it with a silicone layer embedded with YAG:Ce^3+^. An IR camera was used to monitor the surface temperature of the phosphor layer. No heat sink was used to dissipate the heat of the MCPCB; hence, the temperature would increase when the injection current increased.

**Figure 4:**
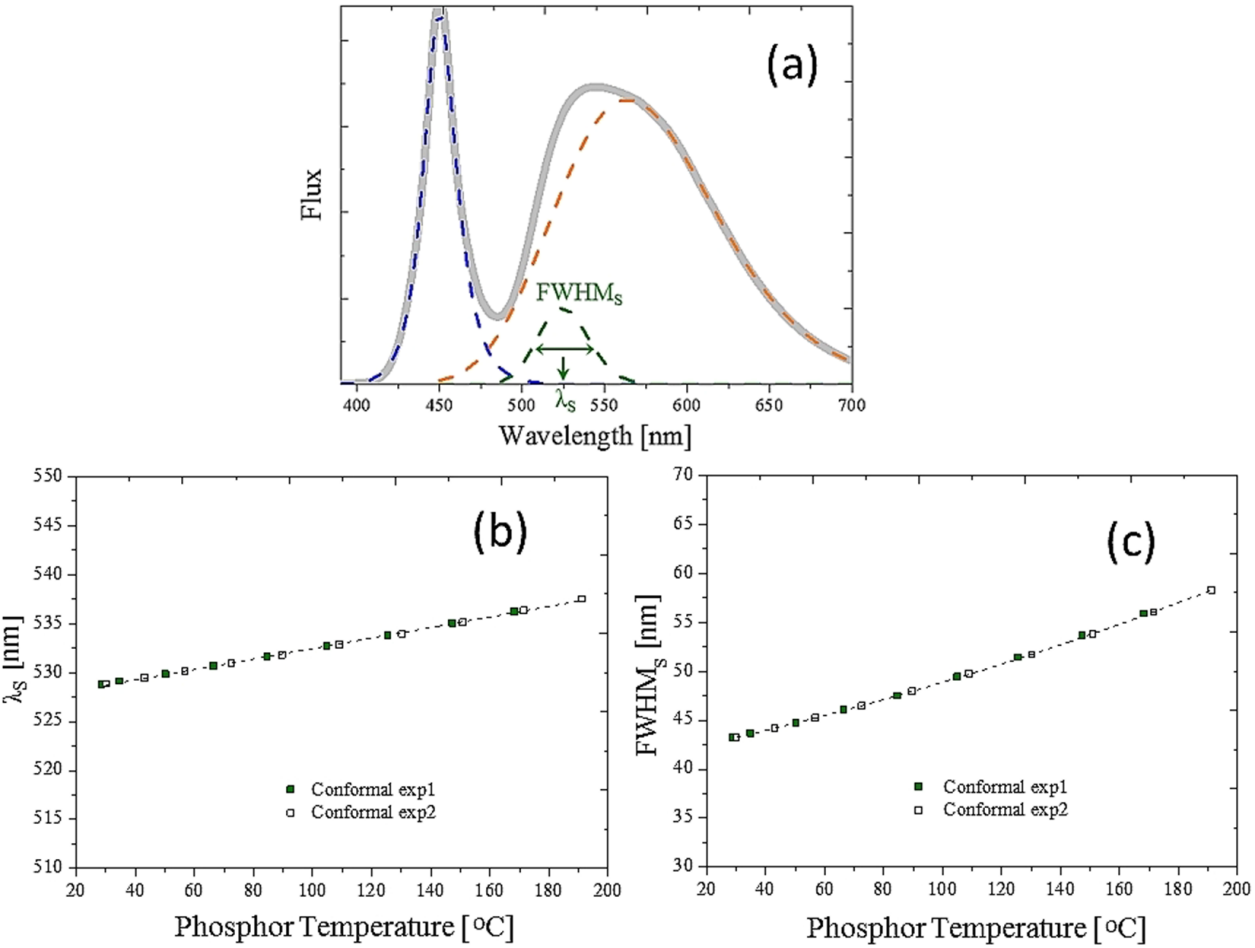
(**a**) Normalized spectrum for the short wavelength band. Correspondence between the (**b**) peak wavelength and (**c**) bandwidth of the phosphor.

**Figure 5:**
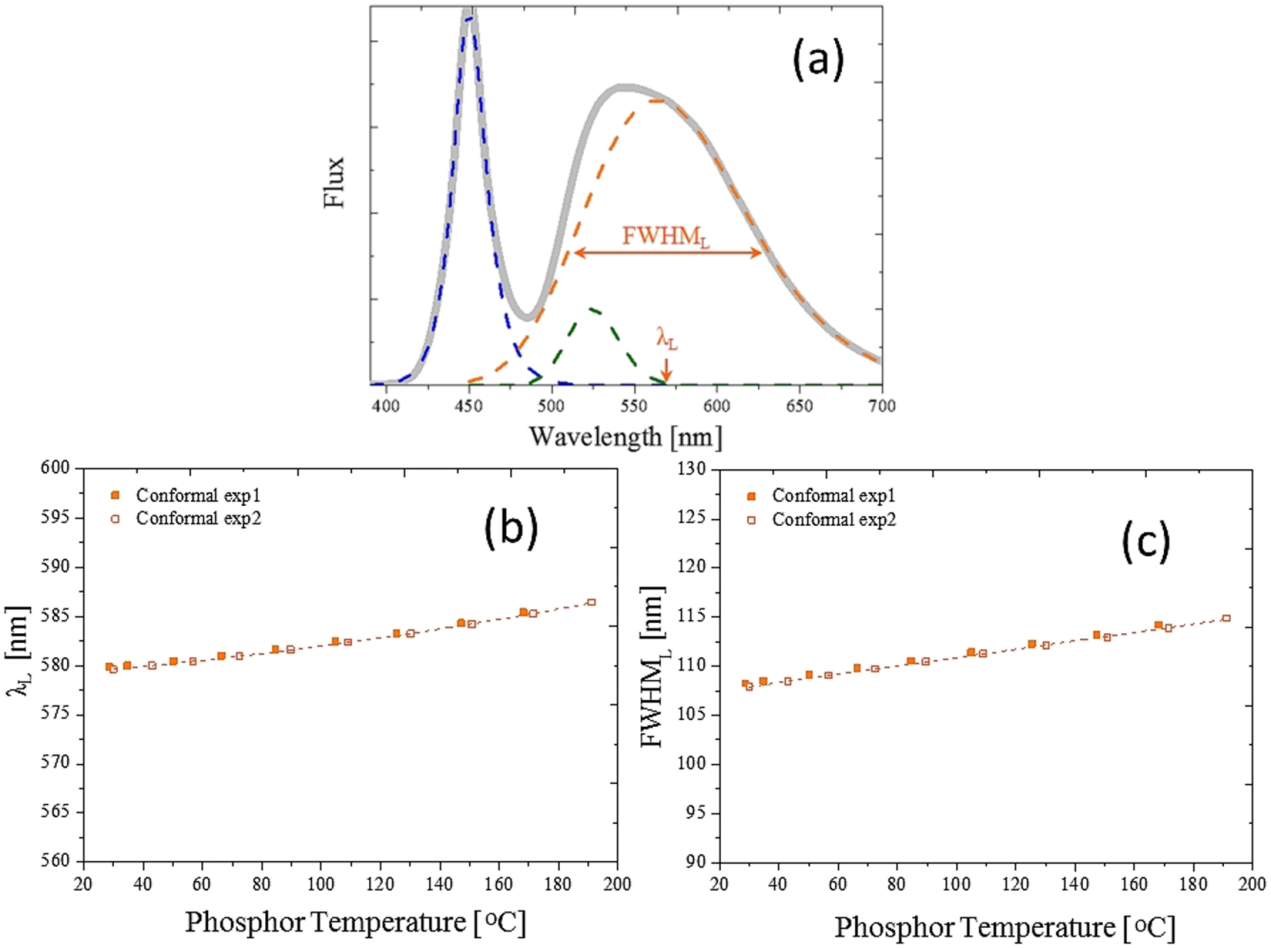
(**a**) Normalized spectrum for the long wavelength band. Correspondence between the (**b**) peak wavelength and (**c**) bandwidth of the phosphor temperature.

The fitting results from the experimental data are illustrated in Figs 4 and 5, where the associations between all four factors are well defined, including the fitting peak wavelengths and the full-width at half-maximums (FWHMs) of the short and long wavelength bands, and the phosphor temperature. This suggests that the phosphor temperature can be precisely measured by checking variations of the emission spectrum.

**Figure 6:**
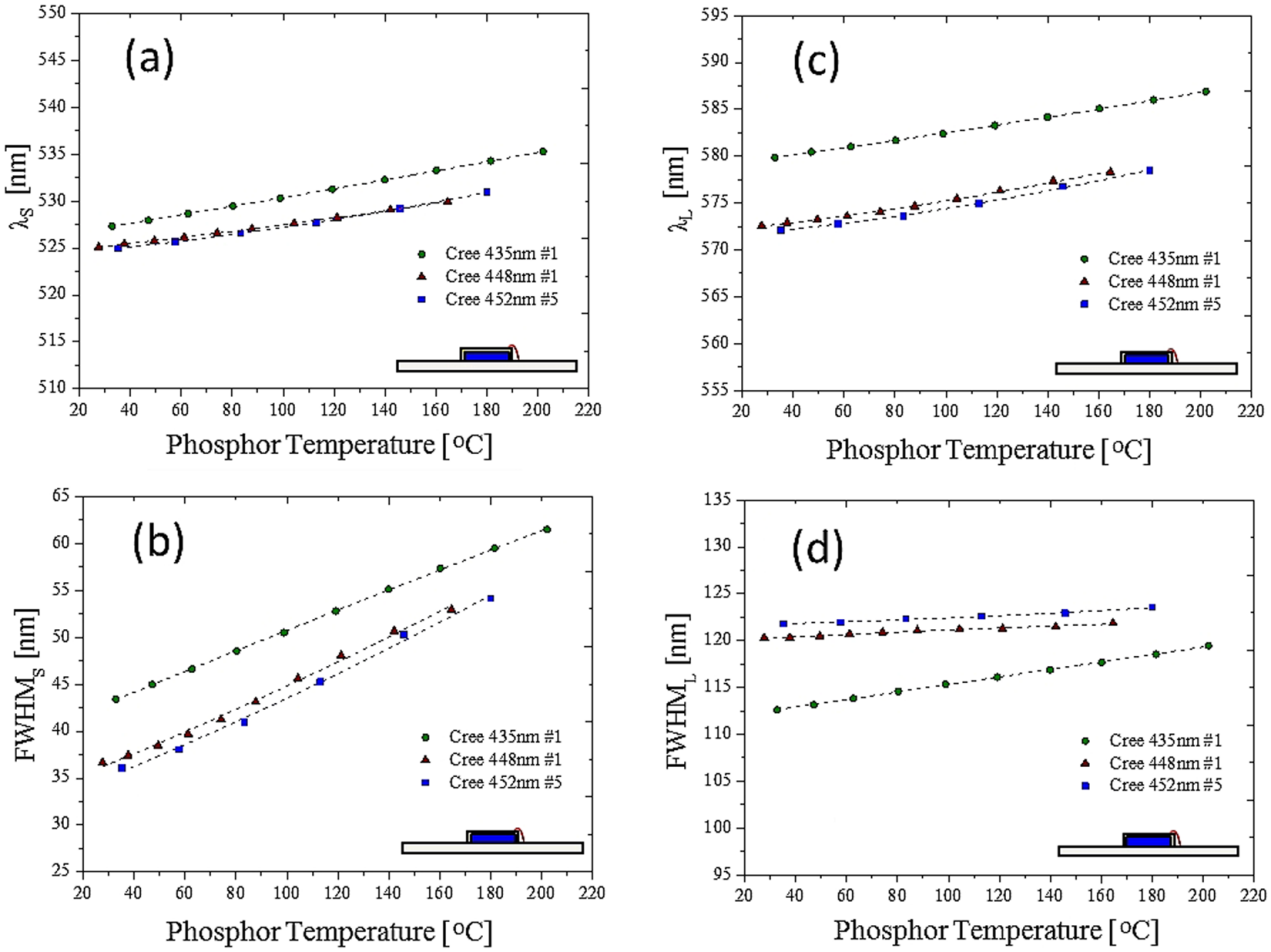
Cases with different blue dies. Correspondence between (**a**) peak wavelength and (**b**) bandwidth and phosphor temperature in the short wavelength band. Correspondence between (**c**) peak wavelength and (**d**) bandwidth and phosphor temperature in the long wavelength band.

**Figure 7:**
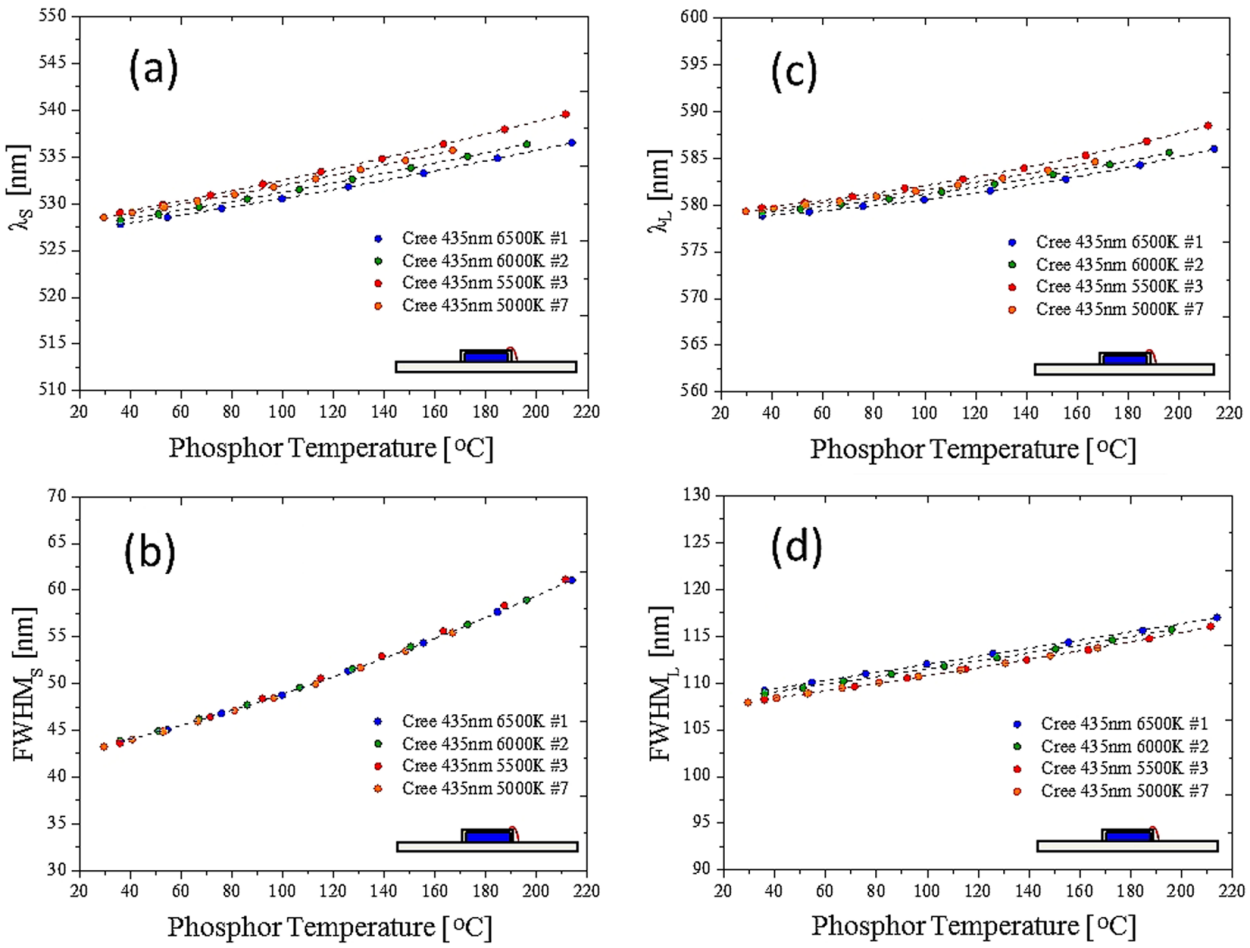
Cases with phosphor concentrations or CCTs. Correspondence between (**a**) peak wavelength and (**b**) bandwidth and phosphor temperature in the short wavelength band. Correspondence between (**c**) peak wavelength and (**d**) bandwidth and phosphor temperature in the long wavelength band.

**Figure 8:**
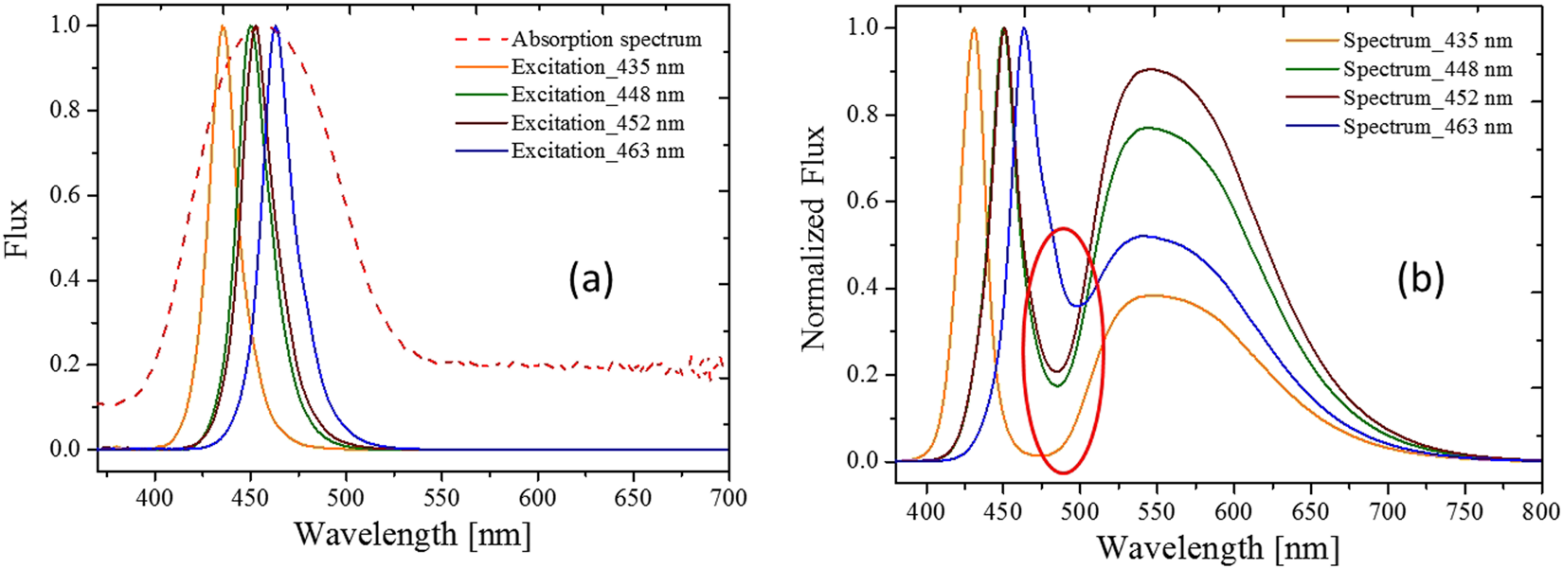
(**a**) Absorption spectrum of the phosphor (dashed line), and the normalized spectra of the blue dies (colored solid lines). (**b**) Normalized spectra of the pc-WLEDs. Red ellipse indicates the parts with large cross-talk.

The results shown in Figs 4 and 5 are for a specific blue die and a specific CCT. The FWHM and the peak wavelength increased with increase in the temperature^33^. The red shift was due to the thermal expansion of the YAG, which increased the crystal field and Stokes shift between the ground state and the excited state. Therefore, regarding this specific design, the phosphor temperature was directly measured by fitting the phosphor normalized emission spectra and one of the four fitting factors that related to the temperature. We endeavored to create a general model for different blue dies with a specific phosphor in different concentrations or CCTs. Figure 6 shows the relationship between the four factors and the phosphor temperature with different blue dies. Figure 7 shows the relationship between the four factors and the phosphor in different concentrations (related to different CCTs). Unfortunately, in the two cases, not all four factors had a single function with respect to the phosphor temperature measured by the IR camera. The absorption spectrum, related to the ${}^{2}{F}_{5/2}-{}^{2}{E}_{g}$ transition of the trivalent cerium, centered at 450 (Fig. 8), explains the inconsistency among different blue dies and phosphor concentrations in Figs 6 and 7. The proposed fitting approach was based on a clear distinction between the blue and yellow spectra. However, when the spectrum of the blue light was near the yellow spectrum, the cross-talk between the two spectra was complicated and could not be neglected. In addition, the reabsorption of the yellow light added further complication. In summary, the part of the yellow spectra close to the blue light was distorted further, becoming worse when the wavelength of the blue light was longer. This result was observed in a study that used green phosphor^34^. However, the recovery of the genuine yellow spectrum without reabsorption is infeasible to apply to this study.

### Precise Detection Model

**Figure 9:**
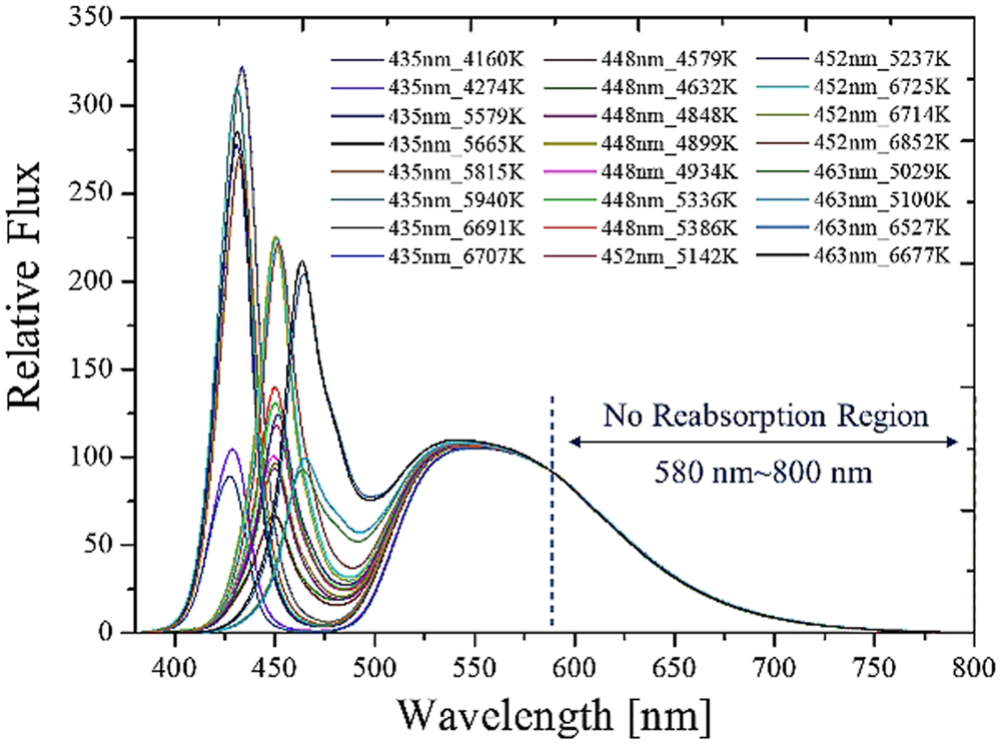
Feature band from 580 to 800 nm is free of cross-talk from the spectra obtained experimental measurement with different blue dies and CCTs. Each line indicates the blue wavelength and the corresponding CCT.

**Figure 10:**
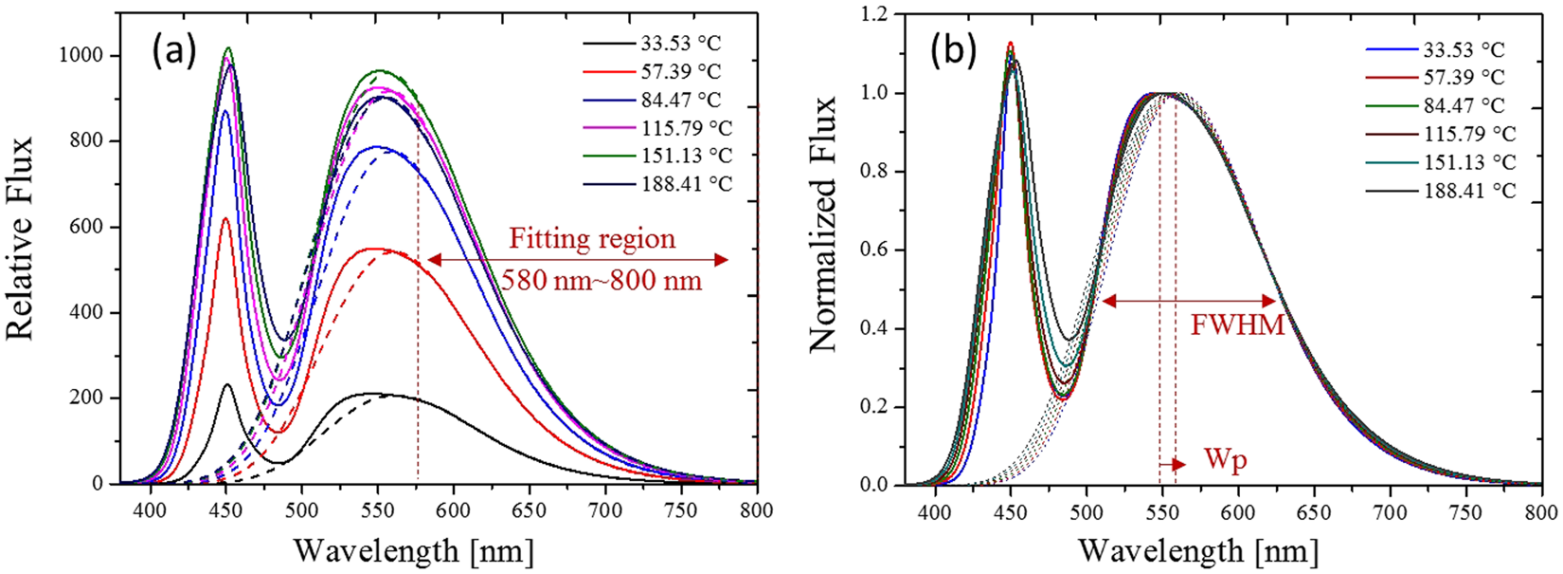
(**a**) Spectra for different phosphor temperatures. (**b**) Normalized spectra of (**a**) with respect to the peak spectral power of the yellow spectra.

An advanced analysis of the spectra with different blue emissions and phosphor concentrations was conducted to find a more effective method for building a simple single function. The method should bear less cross-talk for the crossover range of the short and long bands in the emission spectra away from the blue light. As shown in Fig. 9, the spectra from 580 to 800 nm were almost identical in all cases at room temperature. The spectra from 580 to 800 nm can be regarded the feature band of the entire spectrum. The feature band for different phosphor temperatures is shown in Fig. 10, which clearly shows the temperature effect. Therefore, only one fitting was necessary in that process. A Gaussian function was applied to fit a part of the normalized emission spectrum along the feature band for different phosphor temperatures.

**Figure 11:**
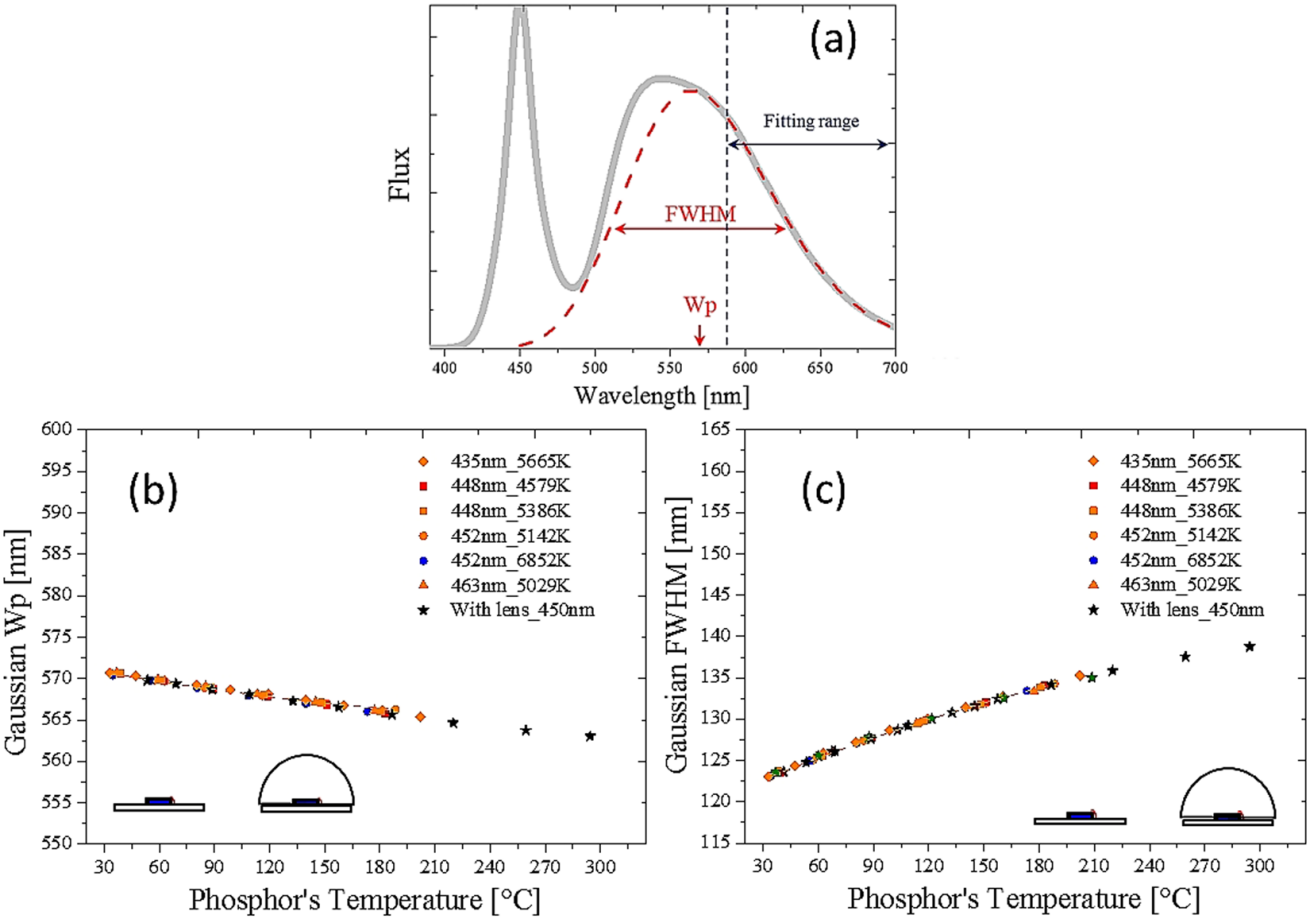
(**a**) Normalized emission spectrum with the fitting band (in red dashed line). Fitted (**b**) peak wavelength and (**c**) the bandwidth for different blue dies and CCTs with and without lens encapsulation.

The peak wavelength (*W*_*p*_) as well as the bandwidth (FWHM) are suitable factors for indicating the phosphor temperature. Various experiments were conducted to collect sufficient data to evaluate the consistency of the fitting factors, as shown in Fig. 11, where various pc-WLEDs were pumped with different blue dies in different phosphor concentrations. Unexpectedly, the fitting single Gaussian function was applicable to the package with and without lens encapsulation. The difference in detected temperatures through the function by the peak wavelength and bandwidth was within 2.5%. We found that the bandwidth part was more accurate than the peak wavelength part because the sensitivity of the bandwidth increased with temperature.

**Figure 12:**
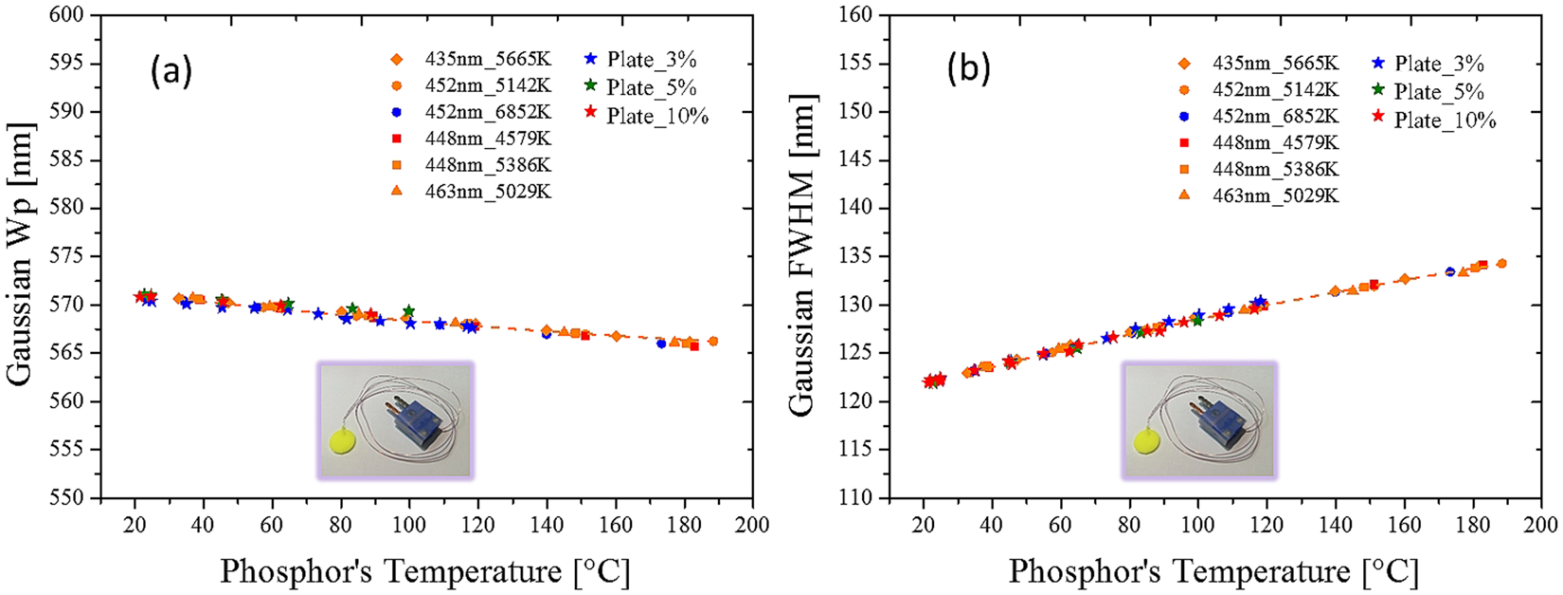
One Gaussian fitting data from a phosphor plate with pc-WLEDs shown in Fig. 10. (**a**) Peak wavelength and (**b**) bandwidth versus the phosphor temperature follows a polynomial of degree 2 (dashed orange lines). Weight concentrations of the phosphor in the plates were 3%, 5% and 10%.

To further evaluate the capabilities and validity of the model, we made a phosphor plate with an approximate thickness of 1 mm. The phosphor embedded in the silicone plate was the same as that used in the conformal-coated pc-WLED, but the silicone plate was far from the blue LED die. A thermocouple was inserted into the phosphor plate to detect the temperature. The phosphor was attached to a copper heating plate to control the temperature, and a blue laser of 450 nm was incident on the plate. An integrating sphere collected the emission light from the phosphor. The experimental data obtained from the conformal-coated pc-WLEDs were quadratic compared with the phosphor temperatures shown in Fig. 12. The proposed method is useful for different pumping light sources and the phosphor plates with various phosphor concentrations.

Using the proposed model, a database was built to obtain the featured quadratic functions for describing the relationships between the fitted peak wavelengths or the fitted bandwidth of the feature band and the phosphor temperatures. Including the calibration of the temperatures of the phosphor layers was essential to build the database. We prepared additional samples of the phosphor plates of conformal coating phosphors on a blue die attached to the substrate. An IR camera detected the phosphor temperatures of the sample surfaces. Once the calibration database was successfully built using the proposed modeling, the derived quadratic function accurately monitored the phosphor temperatures.

**Figure 13:**
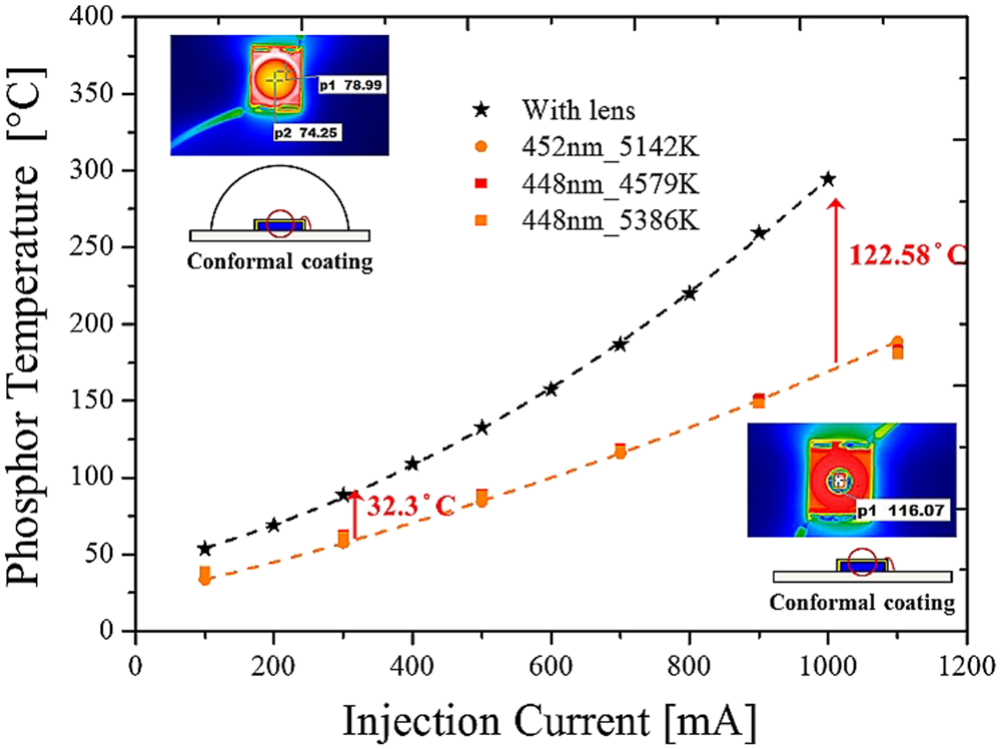
Detection of phosphor temperature for the package with and without lens encapsulation under different injection currents. Black (red) dash line indicates the pc-WLED with (without) lens encapsulation.

Figure 13 shows the experimental measurements, obtained using the proposed detecting model, of the phosphor temperatures with and without lens encapsulation. The conformal coating packaging without lens encapsulation was used to build the database. Then the pc-WLEDs were encapsulated with a silicone lens. The database was used to indicate the temperature of the phosphor layer. The lens encapsulation blocked possible air convection of the phosphor layers; hence, the temperature was higher than the packaging without lens encapsulation. Based on the proposed modeling and the database, we found that the temperature of the sample with lens encapsulation increased by more than 160% compared with the packaging without lens encapsulation under different injection currents. Note that the phosphor temperature as a function of the injection current (Fig. 13) can be adopted only for the same thermal dissipation design.

## Discussion

This study proposed a novel model for precisely measuring the phosphor temperature of a pc-WLED. The detection model can be adopted for different blue dies, phosphor concentrations, phosphor thicknesses, and CCTs, with and without lens encapsulation.

First, this study examined the emission spectra of the phosphor, following which the spectrum was divided into two subbands. Using a Gaussian function to fit the spectra of the subbands, we obtained the fitting peak wavelength and bandwidth, which were well defined for a specific blue die and phosphor concentration. However, inconsistencies were observed when the blue die was at a different peak wavelength and the phosphor was at a different concentration, owing to different cross-talk between the blue and yellow spectra. We found that the normalized spectrum from 580 to 800 nm was almost free from cross-talk. Hence, a new fitting spectrum was made for part of the feature spectrum. The fitting result obtained a peak wavelength, and the bandwidth for the yellow spectrum was without cross-talk. The peak wavelength and the bandwidth corresponded well to the phosphor temperature (temperature deviation <2.5%). Furthermore, the two functions could be fitted using simple quadratic polynomials. Using the two functions, we measured the phosphor temperature under different conditions, including changing the blue die, phosphor concentration, phosphor thickness, and CCT, with and without lens encapsulation. The only restriction was that the phosphor must be the same. These features were considered diverse for phosphors. Hence, the coefficients in the empirical model must be refined individually. However, the proposed method and the procedure should still work well. This empirical model is based on the stable properties of the phosphors. The model cannot correctly predict the phosphor temperature after the phosphors ages. During the ageing stage, the phosphors temperature might not be the most significant factor.

The proposed model based on spectrum fitting can remotely measure the phosphor temperature of a pc-WLED. We successfully demonstrated a correspondence between the yellow spectrum and phosphor temperature from a white light spectrum with large cross-talk. This study provides a highly effective method for monitoring phosphor temperatures, which can be adopted by the sold-state lighting industry to develop more light sources that are reliable and efficient and have high color stability.

## Methods

To measure the phosphor temperature in the experiment, an IR camera took images of the conformal-coated phosphor on a blue die that was attached to a board. Since the phosphor layer was thin, the temperature on the surface of the layer evaluated by the IR camera could be used to indicate the phosphor temperature. The normalized emission spectrum of YAG:Ce^3+^ is shown in Fig. 3, where the yellow phosphor had a peak wavelength at approximately 540 nm. The emission spectrum was caught by a spectrometer in free space or in an integrating sphere. The final approach was based on the feature band of the normalized spectrum as shown in Figs 9 and 10. A Gaussian function was used to fit the feature spectrum so that the equivalent peak wavelength and the bandwidth could be obtained. The experiments were completed at different wavelengths (of the pumping blue light), phosphor concentrations, phosphor thicknesses, with and without lens encapsulation. Both the fitting peak wavelength and bandwidth from the detected spectra were fitted with simple quadratic functions respectively. Subsequently, the fitting functions were used to predict the phosphor temperature in a real pc-WLED.
